# Structural correlates of cognitive impairment in normal pressure hydrocephalus

**DOI:** 10.1111/ane.13052

**Published:** 2018-12-03

**Authors:** Katie A. Peterson, Tom B. Mole, Nicole C. H. Keong, Elise E. DeVito, George Savulich, John D. Pickard, Barbara J. Sahakian

**Affiliations:** ^1^ Department of Psychiatry University of Cambridge Cambridge UK; ^2^ Department of Clinical Neurosciences University of Cambridge Cambridge UK; ^3^ MRC/ Wellcome Trust Behavioural and Clinical Neurosciences Institute University of Cambridge Cambridge UK

**Keywords:** apathy, cognition, neuroimaging, neuropsychology, normal pressure hydrocephalus

## Abstract

**Objectives:**

The pathological bases for the cognitive and neuropsychiatric symptoms in normal pressure hydrocephalus (NPH) have not been elucidated. However, the symptoms may indicate dysfunction of subcortical regions. Previously, volume reductions of subcortical deep grey matter (SDGM) structures have been observed in NPH patients. The present study used automated segmentation methods to investigate whether SDGM structure volumes are associated with cognitive and neuropsychiatric measures.

**Methods:**

Fourteen NPH patients and eight healthy controls were included in the study. Patients completed neuropsychological tests of general cognition, verbal learning and memory, verbal fluency and measures of apathy and depression pre‐ and postshunt surgery. Additionally, patients underwent 3 Tesla T1‐weighted magnetic resonance imaging at baseline and 6 months postoperatively. Controls were scanned once. SDGM structure volumes were estimated using automated segmentation (FSL FIRST). Since displacement of the caudate nuclei occurred for some patients due to ventriculomegaly, patient caudate volumes were also estimated using manual tracing. Group differences in SDGM structure volumes were investigated, as well as associations between volumes and cognitive and neuropsychiatric measures in patients.

**Results:**

Volumes of the caudate, thalamus, putamen, pallidum, hippocampus and nucleus accumbens (NAcc) were significantly reduced in the NPH patients compared to controls. In the NPH group, smaller caudate and NAcc volumes were associated with poorer performance on neuropsychological tests and increased severity of neuropsychiatric symptoms, while reduced volume of the pallidum was associated with better performance on the MMSE and reduced apathy.

**Conclusions:**

Striatal volume loss appears to be associated with cognitive and neuropsychiatric changes in NPH.

## 1 INTRODUCTION

Normal pressure hydrocephalus (NPH) is characterized by a build‐up of cerebrospinal fluid (CSF) in the brain which causes ventriculomegaly despite apparently normal CSF pressure at lumbar puncture. Symptoms include gait apraxia, urinary incontinence and cognitive decline.^1^ In addition, recent research suggests a high prevalence of apathy in NPH patients (65‐86%).^2^, ^3^, ^4^ NPH is treated via surgical CSF diversion which results in clinical and neuropsychological improvement for many patients.^5^

The pathological mechanisms underlying the cognitive and neuropsychiatric symptoms in NPH are not well established. The executive dysfunction in NPH may suggest impaired frontal lobe functioning,^6^ however, there is inconsistent evidence for frontal involvement from imaging studies of regional cerebral blood flow (rCBF) in NPH.^6^, ^7^ The pattern of cognitive decline is consistent with dysfunction of subcortical structures and may indicate disruption to the subfrontal white matter, limbic connections or connections between the frontal lobe and the subcortical deep grey matter (SDGM) structures.^8^ Indeed, impaired rCBF has been found in the periventricular white matter and in the basal ganglia.^7^, ^9^

The presence of apathy (impaired goal‐directed behaviour) in NPH is consistent with fronto‐subcortical pathology. Apathy can result from lesions affecting fronto‐subcortical circuitry,^10^ or from focal lesions of the basal ganglia,^10^ including lesions located in the caudate nucleus.^11^ Diminished caudate volume was previously observed in NPH patients using voxel‐based morphometry and hypothesized to contribute to the clinical symptoms of cognitive impairment and apathy.^12^ However, this association was not examined. We previously found evidence for an association between greater bicaudate ratio (greater ventriculomegaly) with increased levels of apathy and depression in NPH patients.^13^ Additionally, evidence for associations between apathy and rCBF in the anterior cingulate cortices and the right caudate nucleus has been reported.^14^

Subcortical volumes in NPH have not been extensively investigated using quantitative MRI techniques. The periventricular regions may be distorted due to ventriculomegaly. Atrophy may also occur over time due to ischaemia and subsequent cell death.^15^ The few studies that have examined SDGM volumes in NPH focused only on individual structures or did not relate volumetric data to clinical information.^12^, ^16^, ^17^ In the present study, we conducted volumetric assessments of a range of SDGM structures and investigated whether volumes are related to cognitive or neuropsychiatric measures. Subcortical volumes were estimated using automated segmentation techniques. However, in some of the patients there was displacement of the caudate nuclei due to the degree of ventriculomegaly. For this reason, we also used manual tracing techniques to estimate the volume of the caudate in all patients to supplement the analyses based on automated segmentation of the caudate.

## 2 METHODS

### 2.1 Ethics

Ethical approval was obtained from Cambridgeshire 2 Local Research Ethics Committee (LREC No: 06/Q0108/330) as part of a larger project entitled “Functional, Vascular and Structural Correlates of Reversible Dementia in Normal Pressure Hydrocephalus.”.

### 2.2 Participants

Sixteen patients with NPH were recruited between October 2007 and June 2009. Patients gave informed consent prior to enrolling in the study. Patients were assessed and scanned before and a mean of 6.4 months following shunt surgery. Patients were referred to be included in the study by a neurosurgeon (JDP) based on the presence of a clinical picture of gait disturbance, slowing of mentation and/or short‐term memory impairment.^6^ One patient was excluded from the following analyses as they had comorbid Alzheimer’s disease. One additional patient was excluded due to a complicated postoperative clinical course.

Healthy controls were recruited via verbal screening at the CSF multidisciplinary clinic for inclusion criteria, and via advertisement in the local area. Interested partners or spouses of study participants who met the inclusion criteria were also offered healthy volunteer scans. Some healthy volunteers self‐referred after becoming aware of the NPH imaging programme via the patient community. Exclusion criteria for the healthy control group were (i) a diagnosis of a neurological disorder, (ii) the presence of contraindications to MRI scanning and (iii) age <60. Eight healthy controls (HCs) were scanned at one time‐point.

### 2.3 Neuropsychological assessment

Patients completed a brief battery of neuropsychological tests, selected on the basis of their sensitivity to NPH,^5^ pre‐ and postoperatively. Global cognitive functioning was assessed using the Mini‐Mental State Examination (MMSE).^18^ Verbal learning and memory was assessed using the Hopkins Verbal Learning Test (HVLT).^19^ Phonemic and semantic fluency were assessed using the Controlled Oral Word Association Test.^20^ Apathy was assessed using the Cambridge‐developed State Apathy Evaluation scale (self‐rated; AES‐S),^21^ which measures state‐related changes in apathy. Depression was assessed using the short form of the Geriatric Depression Scale (GDS‐15).^22^ IQ was assessed using the National Adult Reading Test.^23^ To minimize practice effects, alternate versions of phonemic and semantic fluency categories and the HVLT word list were used at follow‐up.

### 2.4 MR imaging protocol

MR imaging was performed on a 3 T Siemens Tim Trio using a 12‐channel RF receive coil. The MR imaging protocol included a PDT2, FLAIR, MPRAGE and DTI. The T1‐weighted structural sequence (MPRAGE) was acquired with TR/TE of 2300/2.98 ms, with a resolution of 1x1x1mm.

### 2.5 Image analysis

#### 2.5.1 Automated segmentation

**Figure 1:**
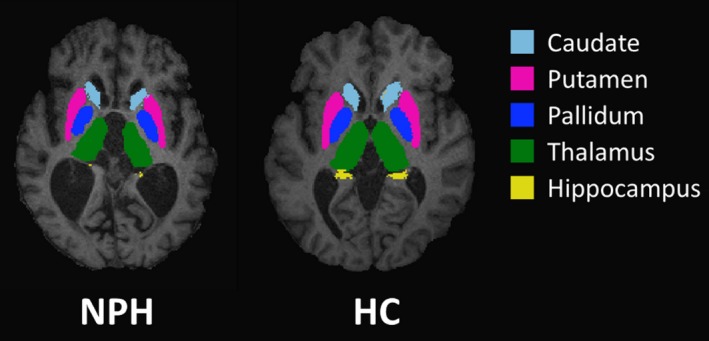
Representative FIRST segmentation of subcortical structures in a patient with normal pressure hydrocephalus (left) and a healthy control subject (right)

Before processing, all images were manually reviewed for artefact. SDGM volumes were estimated for both groups (including at pre‐ and postshunt for NPH patients as the shunt artefact was above the level of the SDGM structures). Subcortical volumes (caudate, nucleus accumbens (NAcc), pallidum, putamen and thalamus) as well as volumes of the hippocampus and the amygdala were extracted from 3D T1‐weighted images using an automated subcortical registration and segmentation tool in FSL (FSL FIRST; www.fmrib.ox.ac.uk/fsl). Examples of subcortical segmentation are shown in Figure 1. All automated segmentations were thoroughly individually reviewed for accuracy. SDGM volumes were normalized for head size by multiplying by the volumetric scaling factors obtained using SIENAX,^24^ part of FSL.^25^ SIENAX starts by extracting brain and skull images from the single whole‐head input data.^26^ The brain image is then affine registered to MNI152 space (using the skull image to determine the registration scaling)^27^; primarily in order to obtain the volumetric scaling factor to be used as a normalization for head size. Next, tissue‐type segmentation with partial volume estimation is carried out,^28^ in order to calculate total volume of brain tissue. As laterality effects were not hypothesized, normalized right and left volumes of the SDGM structures were combined to yield a single total volume for each structure.

#### 2.5.2 Caudate tracing

**Figure 2:**
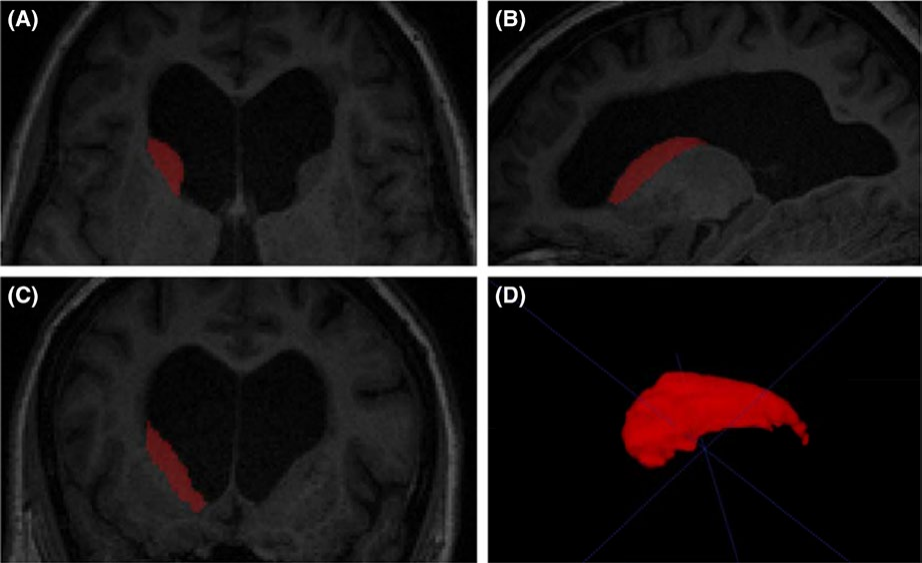
Segmented right caudate in (A) axial section, (B) sagittal section and (C) coronal section in a representative NPH patient; (D) 3D rendering of caudate volume

Manual tracing was performed on T1‐weighted images using ITK‐SNAP version 3.4.0 (www.itksnap.org). 29 Right and left caudate nuclei were traced on contiguous axial slices, as described by Looi et al,^30^ beginning with the most inferior slice where the caudate nucleus was visible, bounded by the frontal white matter, the anterior commissure and the internal capsule.^30^ Tracing proceeded superiorly from the head to the tail of the caudate until the tail was no longer distinguishable from the wall of the lateral ventricle. Caudate segmentation was then checked in the coronal and sagittal views where any errors were corrected. Finally, a 3D view of the segmented caudate nucleus was compiled and checked for abnormalities (Figure 2). Volumes were normalized for head size by multiplying by the volumetric scaling factors from SIENAX. Normalized right and left caudate volumes were combined to yield a single total volume.

### 2.6 Statistical analysis

Data were analysed using the Statistical Package for the Social Sciences (SPSS) software version 25 (IBM Corp, Armonk, NY). Independent samples *t* tests were conducted to test for group differences in age and SDGM volumes. Additionally, ANCOVAs were conducted to test for group differences in SDGM volumes while controlling for age and sex. Pearson’s *r *correlations were conducted to investigate associations between demographic data, volumetric data and neuropsychological test scores. However, where variables violated assumptions of normality, Spearman’s correlation coefficient (*r_s_*) was used. Paired samples *t* tests were conducted to test for pre‐ to postshunt differences in SDGM volumes and neuropsychological test scores.

Pearson’s *r* correlations investigated associations between caudate volumes obtained via automated segmentation and via manual tracing. Differences in the volumes obtained via the two methods were investigated using paired samples *t *tests. Independent samples *t* tests were conducted to investigate differences in volumes between patients included in or excluded from the automated segmentation analyses.

## 3 RESULTS

### 3.1 Demographics

The final patient group consisted of 14 patients (9 male, 5 female). Baseline mean (SD) age was 76.4 (3.9), range = 70‐84, IQ was 110.8 (10.4), MMSE was 24 (3.3), and years of education was 11.1 (2.2). Individual patient details are shown in Table 1. Two patients had comorbid depression (patients 6 and 9 in Table 1). Four HCs were male. The NPH group was significantly older than HCs (HC group mean (SD) age = 70.3 (6.6), range = 64‐80); (*t* (9.89)  = 2.43, *P* = 0.036).

**Table 1:** Normal pressure hydrocephalus patient demographics

Patient No.	Age	Sex	IQ	Years of education	Time to retest	Aetiology	Gait disturb	Urinary symptoms	MMSE Pre	MMSE Post
**1**	84	F	122	11	7	Idiopathic	+	+	26	28
**2**	78	F	100	11	7	Idiopathic	+	−	18	21
**3**	75	M	103	11	8	Idiopathic	+	+	23	22
**4**	77	M	112	14	7	Idiopathic	+	+	26	27
**5**	78	M	n/a	13	5	Idiopathic	+	+	n/a	28
**6**	78	M	n/a	9	5	Idiopathic	+	+	20	15
**7**	72	M	87	10	7	Query aqueduct stenosis	+	+	18	26
**8**	82	M	118	9	7	Secondary NPH	+	+	24	25
**9**	71	M	116	16	5	Idiopathic	+	+	25	28
**10**	70	F	116	15	11	Idiopathic	+	‐	27	28
**11**	74	F	109	12	8	Idiopathic	+	+	26	26
**12**	77	M	115	9	5	Idiopathic	+	+	27	28
**13**	76	M	107	8	<1	Idiopathic	+	+	26	22
**14**	78	F	124	12	8	Idiopathic	+	n/a	26	27

n/a, information not available.

### 3.2 Automated segmentation

The following analyses are based on automated subcortical segmentation. While this technique avoided observer bias, it reduced the sample size because of gross displacement caused by ventriculomegaly of all SDGM structures in three patients and displacement of the caudate nuclei in an additional three patients at pre‐shunt. At postshunt, there was displacement of all structures in two patients and displacement of the caudate nuclei in a further two patients. As a result of this, both pre‐ and postshunt volumetric data for each structure were available for a limited number of patients. Therefore, correlational analyses were conducted at two time‐points (pre‐ and postshunt) rather than on pre‐ to postshunt change scores.

Automated segmentation was successful in the HCs and all remaining patients so that analyses of SDGM volumetric data is based on 11 patients preoperatively and 12 postoperatively (apart from data for caudate volume which is based on eight patients preoperatively and 10 postoperatively).

#### 3.2.1 Subcortical volumes

Mean volumes of the SDGM structures for both groups are shown in Table 2. Volumes of all SDGM structures except the amygdala were significantly smaller in the NPH group at both pre‐ and postshunt compared to controls. These remained significant after controlling for age and sex.

**Table 2:** Group differences in SDGM volumes at pre‐ and postshunt

SDGM structure	Control Mean (SD) volume	NPH Pre‐shunt Mean (SD) volume	NPH Post‐shunt Mean (SD) volume
Caudate	8627.6 (726.8)	6720.4 (485.1)^a^	6965.3 (822.6)^a^
Thalamus	17590.6 (1865.1)	13141.5 (883.4)^a^	14112.4 (1287.1)^a,b^
Putamen	11658.7 (1269.2)	7150.3 (1771.8)^a^	8737.2 (1410.2)^a^
Pallidum	4139.9 (562.4)	2903 (639)^a^	3226.5 (646.5)^a^
Hippocampus	9211.9 (1271.5)	5356.7 (1142.8)^a^	6302.9 (1051.6)^a,b^
Amygdala	3148.5 (638.9)	2631.4 (605.9)	2889.4 (642.2)
Nucleus accumbens	1010.6 (237.9)	483.67 (100.9)^a^	531.04 (182)^a^

Volumes presented (including caudate volume) are taken from the automated segmentation analysis, and are in mm^3^

a Significant volume difference compared to controls, *P < *0.05

Significant volume difference compared to pre‐shunt, *P* < 0.05.

In the NPH group, volumes of the thalamus, *t* (10)  = −2.85, *P* = 0.017; and hippocampus, *t* (10) = −3.02, *P* = 0.013, were significantly increased following shunt surgery.

#### 3.2.2 Neuropsychological profile pre‐ and postshunt surgery

Neuropsychological test scores for the NPH patients are shown in Table 3. Performance on semantic fluency, HVLT immediate and HVLT learning was significantly improved following shunt surgery. In addition, there was a trend for improvement in all remaining test scores, except for GDS‐15.

**Table 3:** Neuropsychological outcome following shunt surgery

Test	N	Pre‐shunt Mean (SD)	Postshunt Mean (SD)	*t*	*df*	*P*
MMSE	12	23.92 (3.40)	24.58 (3.92)	−0.70	11	0.497
AES‐S	10	18.60 (7.95)	15.60 (9.06)	1.15	9	0.278
GDS‐15	10	5.10 (2.51)	5.10 (3.93)	0.00	9	1.000
Phonemic fluency	12	29.75 (14.20)	30.33 (16.96)	−0.19	11	0.853
Semantic fluency	11	10.00 (3.98)	13.45 (3.83)	−2.40	10	0.038*
HVLT immediate	12	3.83 (1.40)	4.58 (1.68)	−3.00	11	0.012*
HVLT learning	12	13.83 (4.86)	17.50 (6.07)	−2.70	11	0.020*
HVLT delayed	12	2.17 (2.76)	3.08 (4.17)	−0.74	11	0.473

MMSE, Mini‐Mental State Examination; AES‐S, self‐rated State Apathy Evaluation; GDS‐15, Geriatric Depression Scale short form; HVLT, Hopkins Verbal Learning Test

* *P < *0.05.

#### 3.2.3 Correlation of volumetric data with neuropsychology scores

The significance value for the Shapiro‐Wilk test was less than 0.05 for the following: pre‐shunt MMSE score, pre‐shunt semantic fluency score, pre‐shunt HVLT delayed score, postshunt MMSE score, postshunt AES‐S and postshunt HVLT delayed score, suggesting that these data are not normally distributed. As such, Spearman’s correlation coefficient (*r_s_*) was used to investigate associations with these variables.

##### Pre‐shunt

NAcc volume was significantly positively correlated with HVLT immediate (*r* = 0.70, *P* = 0.035). That is, greater volume was associated with better performance. There were no other significant correlations between SDGM volumes and cognitive test scores at pre‐shunt.

##### Postshunt

Caudate volume was significantly positively correlated with MMSE score (*r_s_ *= 0.65, *P* = 0.040), and semantic fluency (*r* = 0.67, *P* = 0.035), and significantly negatively correlated with AES‐S (*r* = −0.83, *P* = 0.006). NAcc volume was significantly positively correlated with MMSE (*r_s_* = 0.78, *P* = 0.002), HVLT immediate (*r* = 0.58, *P* = 0.039), HVLT learning (*r* = 0.68, *P* = 0.011) and HVLT delayed (*r_s_* = 0.63, *P* = 0.022); and significantly negatively correlated with AES‐S (*r_s_* = 0.81, *P* = 0.001). For the above significant positive correlations, greater volumes were associated with better cognitive test performance and reduced apathy. Volume of the pallidum was significantly negatively correlated with MMSE score (*r_s_* = −0.55, *P* = 0.050) but significantly positively correlated with AES‐S (*r_s_* = 0.65, *P* = 0.023). That is, greater pallidum volume was associated with poorer performance on the MMSE and increased apathy.

### 3.3 Caudate tracing

Automated segmentation of the caudate failed for a number of the patients, reducing the sample size and meaning that pre‐ and postshunt correlations with clinical measures were based on different participant Ns. Therefore, manual caudate tracing was conducted to further examine the relationship between caudate volume and clinical scores in the patient group.

Manual tracing was conducted for all 14 patients at pre‐ and postshunt. Caudate volumes obtained via manual tracing and automated segmentation were significantly correlated at both pre‐ (*r* = 0.74, *P* = 0.035) and postshunt (*r* = 0.76, *P* = 0.011). Mean manually traced caudate volumes (pre‐shunt mean (SD) = 6288.7 (1386.2) mm^3^; postshunt mean (SD) = 5610 (901.5) mm^3^; N = 14) were smaller than those obtained via automated segmentation (pre‐shunt mean (SD) = 6720.4 (485.1), N = 8; postshunt mean (SD) = 6965.3 (822.6), N = 10). This difference was statistically significant for the postshunt volumes only, *t* (9) = 6.90, *P* < 0.001. Patients were split according to whether they were included in or excluded from (due to unsuccessful caudate segmentation) the automated segmentation analyses. The mean manually segmented caudate volumes did not differ significantly between the two groups at pre‐ (*P* > 0.05) or postshunt (*P* > 0.05). Therefore, the smaller volumes obtained via manual tracing are not due to the inclusion of additional participants.

#### 3.3.1 Correlation of volumetric data with neuropsychology scores

##### Pre‐shunt

Manually traced caudate volumes were significantly positively correlated with MMSE score (*r* = 0.63, *P* = 0.022), phonemic fluency (*r* = 0.64, *P = *0.019), semantic fluency (*r = *0.78, *P* = 0.003) and HVLT delayed (*r* = 0.59, *P = *0.035). That is, greater caudate volume was associated with better performance for all four tests.

##### Postshunt

At postshunt, manually traced caudate volumes were significantly positively correlated with MMSE score (*r = *0.78, *P = *0.001), meaning greater caudate volume was associated with better performance. Additionally, caudate volume was significantly negatively correlated with AES‐S (*r *= −0.69, *P* = 0.009) and GDS‐15 (*r = *−0.55, *P* = 0.042), meaning greater caudate volume was associated with reduced apathy and depression.

## 4 DISCUSSION

We conducted a volumetric assessment of subcortical structures in NPH patients using automated segmentation. Manual tracing was conducted to supplement the caudate volume data. Caudate volumes obtained via automated and manual segmentation were significantly correlated at both pre‐ and postshunt suggesting that automated segmentation was satisfactory for the majority of patients.

All of the SDGM structures apart from the amygdala were significantly reduced in the NPH patients compared to healthy controls, and remained so after adjusting for age and sex. We conducted correlations to investigate associations between SDGM structure volumes and neuropsychological test performance. Using the data derived from automated segmentation, only NAcc volume was significantly associated with HVLT immediate score at pre‐shunt; while at postshunt, larger caudate and NAcc volumes were associated with better performance on the MMSE, and reduced self‐rated apathy. Caudate volume was also positively associated with semantic fluency at postshunt; and NAcc with verbal learning and memory. In addition, greater volume of the pallidum at postshunt was associated with poorer performance on the MMSE and increased self‐rated apathy. The association between pallidum volume and scores is in the unexpected direction and should be clarified in a larger sample.

Since the analyses relating to caudate volume were based on small (and differing) Ns manual caudate tracing was also conducted. Larger manually traced caudate volumes were associated with better performance on the MMSE, verbal fluency and the HVLT delayed subtest at pre‐shunt. At postshunt, larger volumes were associated with better performance on the MMSE as well as reduced self‐rated apathy and depression. These results suggest that volume of the caudate and the NAcc in patients with NPH is associated with the cognitive and neuropsychiatric symptoms.

The caudate nucleus and the NAcc are two important structures within the striatum which play important roles in movement, cognition, learning and motivation or goal‐directed behaviour.^31^, ^32^, ^33^ The striatum is linked to the frontal cortex via parallel frontal‐subcortical circuits, lesions to which can cause executive dysfunction, personality changes and impaired motivation or apathy.^10^, ^34^ Reduced pre‐operative caudate volume in NPH patients was previously noted,^12^ and hypothesized to contribute to cognitive decline and apathy. The nature of the correlations in the present study indicated that reduced caudate and NAcc volume was associated with increased apathy, supporting the notion that dysfunction of these regions underlies apathy in NPH. Further, Kanemoto et al.^14^ reported evidence for an association between reduced apathy following shunt with improved rCBF in the anterior cingulate cortices and the right caudate nucleus. Therefore, volume changes of subcortical regions may be related to changes in rCBF. Taken together, these results provide evidence for the importance of striatal volume loss in the cognitive and behavioural symptoms in NPH and might suggest that at least some of the cognitive decline in patients could be due to subcortical dysfunction.

An important limitation of our study concerns the significant age difference between the patients and the controls in our sample. We cannot rule out the possibility that the smaller SDGM structure volumes seen in the patient group are due to the age difference compared to controls. However, the difference in volumes between the healthy controls and the NPH patients ranges from −16% to −52% which is substantially greater than would be expected according to published estimates of age‐related decline of subcortical structures.^35^ Therefore, while some of the group difference may be explained by the age difference, it is unlikely that the age difference can account for the majority of the group differences. While we have attempted to control for the age difference, a further study with more closely matched patients and controls will help to clarify this issue. Another limitation concerns the small sample sizes, and the fact that some of the sample sizes were further reduced due to the limitations of automated segmentation methods in this patient group. We attempted to overcome this issue by incorporating manual tracing to supplement the volumetric data obtained using automated segmentation. However, this study requires replication in the future using larger sample sizes. Finally, our results should be interpreted with caution, as we have not corrected for multiple comparisons since this is a small pilot study. Indeed, the unexpected direction of the correlations between the volume of the pallidum with test scores might suggest that some of our significant associations reflect a type I error. However, the associations relating to caudate and NAcc volume are consistent in terms of direction, and warrant further investigation.

In summary, while most of the SDGM structures investigated in the present study were significantly smaller in NPH patients compared to controls, striatal volume loss appears to be associated with the cognitive and neuropsychiatric symptomology in NPH. The results confirm our earlier hypothesis that reduced caudate volume is associated with increased apathy, and may implicate the striatal regions in cognitive and motivational impairments experienced by patients with NPH.

## CONFLICTS OF INTERESTS

Barbara J. Sahakian consults for Cambridge Cognition, Peak (Brainbow), Mundipharma and has share options in Cambridge Cognition. John D. Pickard reports grants from NIHR Senior Investigator Award and grants from NIHR Cambridge Brain Injury HTC. All other authors have nothing to declare.
